# Chinese acute ischemic stroke treatment outcome registry (CASTOR): protocol for a prospective registry study on patterns of real-world treatment of acute ischemic stroke in China

**DOI:** 10.1186/s12906-017-1863-4

**Published:** 2017-07-06

**Authors:** Weiping Sun, Qianhua Ou, Zhijun Zhang, Jiazhi Qu, Yining Huang

**Affiliations:** 1 Department of Neurology, Peking University First Hospital, No.8, Xishiku Street, Beijing, 100034 China; 2 Department of medical affairs, Techpool Bio-Pharma Co., LTD, Beijing, 100022 China

**Keywords:** Acute ischemic stroke, Cost-utility, Outcome, Cost-effectiveness, Protocols, Registry study, Therapy

## Abstract

**Rationale:**

Stroke presents a serious health problem in China. Despite progresses made in recent years, there is still a lack of effective treatments for acute ischemic stroke (AIS) in clinical practices.

**Aims:**

The Chinese Acute Ischemic Stroke Treatment Outcome Registry (CASTOR) is designed to evaluate the patterns and cost-effectiveness of current treatments for AIS in real-world settings in China.

**Design:**

CASTOR is a prospective, multi-center study registered with ClinicalTrials.gov (NCT02470624) with a target sample size of 10,000 patients who are experiencing AIS. The patients are treated for AIS following the Chinese stroke guideline and local practice. Real-world data on treatment regimens, outcomes and costs are collected at baseline (Visit 1) and during subsequent visits (Visit 2 to Visit 5) after medication treatments.

**Outcome:**

The primary objective of the present study is to analyze the current treatment status of AIS in real world settings. The secondary objectives include: 1) to compare the effectiveness of common treatment regimens, 2) to analyze the cost-effectiveness of different treatment regimens for AIS, 3) to analyze the incidence of adverse events and complications in enrolled patients with AIS, 4) to analyze the effect of Trial of Org 10,172 in Acute Stroke Treatment (TOAST) classification on the specific therapies during acute phase treatment period.

**Discussion:**

In face of changing treatment patterns and increasing demand from medical insurers for cost-effectiveness data in China, a large-scale registry study examining the real-world patterns of AIS in hospitals is needed. The CASTOR study will help to find favorable cost-utility treatment regimens for AIS and improve the overall treatment outcome of Chinese patients with AIS.

**Electronic supplementary material:**

The online version of this article (doi:10.1186/s12906-017-1863-4) contains supplementary material, which is available to authorized users.

## Background

Stroke is one of the leading causes of death and long-term disability worldwide [1] and in China [2, 3]. As the most common type of stroke, acute ischemic stroke (AIS) accounts for approximately 60% to 80% of all strokes [4]. An epidemiological population-based study in Germany revealed that ischemic stroke can be classified several subtypes with different incidence rates and should not be regarded as a homogeneous disease condition [5]. In China, more than 7,000,000 patients are suffering from stroke and 2,000,000 new cases of stroke are diagnosed and stroke-associated deaths account for 20% of total mortality every year [6, 7]. Finding an effective treatment for stroke is thus a national priority in China [8].

For drug treatment of ischemic stroke, only thrombolytic medications and antiplatelet agents are supported by strong clinical evidences at present [9, 10]. An observational study with 6483 patients demonstrated that intravenous alteplase is safe and effective for suitable patients within 3 h of stroke onset [11]. However, their applications are often limited by a myriad of factors such as a narrow time window of treatment and a potential risk of cerebral bleeding, resulting in low usage in real-world practices. For example, the percentage of patients with stroke who are receiving thrombolytic therapy is less than 3.5% in the US [12] and 1% to 3% in China [13]. In patients with ischemic stroke treated with tissue plasminogen activator (tPA), disturbances of consciousness and increasing age are associated with increased in-hospital mortality [14]. Several trials confirm the safety and efficacy of combined intravenous thrombolysis and thrombectomy after AIS [15, 16]. The endovascular treatment is significantly restricted by equipment, cost and experienced physicians [17–20]. Overall, there is still a lack of effective treatment for stroke.

In the last two decades, ‘real-world’ population-based stroke registries in US, European Union, Australia, New Zealand defined global standards [11, 14] have help us to improve the management of stroke. The China National Stroke Registry (CNSR) study evaluated the pattern of then current medical practices and quality of care delivery for stroke patients at the national level in the previous decade. As one of the largest cohort studies on AIS in China, the CNSR enrolled more than 20,000 subjects from 2007 to 2008 [21] and several publications have come out based on that registry study [22–26]. However, data collected in CNSR are not suitable for evaluating the outcome of different treatments due to only a few treatment categories captured in CNSR. Since CNSR, the real-world treatment patterns for AIS in China have evolved and the demand from Chinese medical insurers for cost-effectiveness data has increased. Important questions remain to be solved. For example, to date, more than 100 drugs for the treatment of AIS have been approved by the China Food and Drug Administration (CFDA), which are widely used in clinical practice. However, there are no large-scale clinical studies that compare the therapeutic effects and the cost-utility of different treatments in AIS. Therefore, CASTOR is designed to evaluate the current real-world treatments for AIS. It is expected that the results of CASTOR will be used to guide the clinical practice for AIS treatment in China.

## Methods

### Study design

This study is a prospective, multi-center registry involving 40 tertiary hospitals in China. The hospitals in our study are required to have a neurology ward and CT or MRI equipment. The admitted stroke patients should be over 100 cases per year. The study protocol has been approved by the Ethics Committee of all participating hospitals. The planned enrollment is 10,000 patients with AIS as defined in the current (2014) Chinese Guidelines for the Diagnosis and Treatment of Acute Ischemic Stroke [27]. A total of 5 visits within a 1-year follow-up period will be carried out. Demographic data at baseline, the therapeutic effect, cost-utility, adverse events and complications with different treatments will be analyzed. The influence of TOAST classification on the specific therapy regimens in AIS will be also evaluated. All study conducts will follow the provisions of the ICH GCP and the Declaration of Helsinki. The study is registered with ClinicalTrials.gov (NCT02470624).

### Patient population

All patients are recruited in the study voluntarily and should have signed the informed consent. The study inclusion criteria and exclusion criteria are summarized in Table 1. Patients can be withdrawn from the study at the investigator’s discretion, if it is to his/her best interests. An exit evaluation should be conducted for each withdrawn patient and the reason for withdrawal should be recorded in the electronic case report form (eCRF).

**Table 1:** Inclusion and Exclusion Criteria

Inclusion criteria
1. Age ≥ 18 years
2. Acute ischemic stroke diagnosed according to the Chinese Guideline for Diagnosis and Treatment of Ischemic Stroke (2014)
3. Admitted within 1 week after onset of stroke or within treatment time window for patients with thrombolysis therapy
4. Consent to participation in this study
Exclusion criteria
1. Patients with cerebral hemorrhage confirmed by CT imaging or MRI
2. Patients complicated by serious systemic diseases with an expected survival of less than 3 months
3. Patients cannot provide continuous follow-up information

Note: Computed tomography (CT), Magnetic resonance imaging (MRI)

## Interventions

### Specific treatment

The specific treatments against ischemic stroke as recommended by the Chinese Guidelines for the Diagnosis and Treatment of Acute Ischemic Stroke (2014) [27] will be selected by the investigators according to the symptoms and medical history of the patients. The commonly used drugs for AIS in China include: intravenous thrombolysis drugs, antiplatelets, anticoagulants, drugs for volume expansion, neuroprotectants, medicines for improving the cerebral blood flow, traditional Chinese medicine.

### Concomitant medications

Concomitant medications refer to the treatments non-specific for AIS (i.e., drugs for the treatment of comorbidities) that the patients receive during the study duration, such as dehydrants, antihypertensive drugs, antihyperlipidemic drugs, hypoglycemic drugs, and anti-infective drugs.

## Data collection

**Fig. 1:**
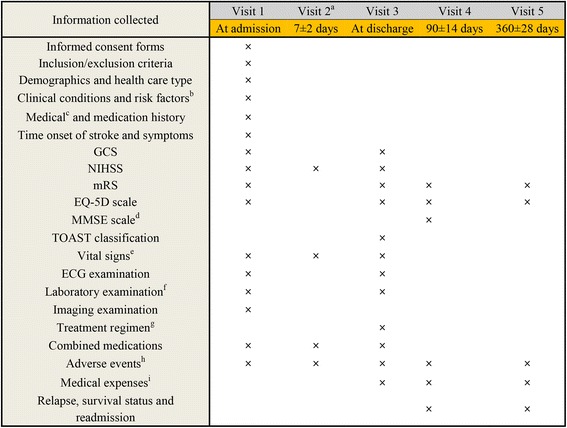
Study schedule of assessments. GCS, Glasgow coma Scale; NIHSS, National Institutes of Health Stroke Scale; mRS, Modified Rankin Scale; EQ-5D, EuroQol-5 Dimensions; MMSE, Mini-mental state examination; TOAST, Trial of Org 10,172 in Acute Stroke Treatment; ECG, Electrocardiograph. a. Visit 2 could be skipped if the patient is discharged at that time; b. optional items; c. It includes stroke, hypertension, diabetes, dyslipidemia, coronary heart disease, atrial fibrillation, carotid plaques, and tumors; d. MMSE is available for outpatient visits of visit 4; e. It includes body temperature, pulse, respiration and blood pressure, and body temperature should be collected at admission and 24 h after admission at visit 1; f. It includes examinations of blood routine test, blood glucose, blood lipids, liver and renal functions, myocardial enzymes, serum electrolytes, and coagulation function; g. It refers to the specific treatments for AIS, and the non-specific treatments will be recorded in the item “combined medications”; h. It is recorded from the time of obtaining the signed informed consent form to 30 days after the last administration; i. It refers to the direct costs, including the direct medical costs and the direct non-medical costs

All data will be collected using eCRFs and an online electronic data capture (EDC) system, in line with the current clinic routine. Contributors to the registry will be responsible for data entry and cleaning. Data will be collected at baseline (Visit 1, at the time of recruitment) and subsequent visits (Visit 2, 7 ± 2 days after medication treatment; Visit 3, at hospital discharge; Visit 4, 90 ± 14 days after medication treatment, and Visit 5, 360 ± 28 days after medication treatment). The study schedule of assessments is as shown in Fig. 1. We will share with others the identified individual-patient data if the results will be published.

## Outcome assessments

To align data collection with therapeutic efficacy evaluation, an outline of visits has been developed and an *outcome assessment analysis* will be carried out. The primary efficacy variable of this study is the percentage of patients with mRS =0–2 at Visit 4. Secondary efficacy variables include the percentage of patients with poor prognosis (mRS =3–5) and death (mRS =6) at Visit 4, the change from baseline in EuroQOL five dimensions questionnaire (EQ-5D) VAS at Visit 3, the frequency of adverse events and complications in enrolled patients with AIS, and the change from baseline in National Institutes of Health Stroke Scale (NIHSS) total score at Visit 3 according to different TOAST classification.

A *Cost-utility analysis* will be conducted for the different treatments. The quality-adjusted life year (QALY) is used as the measure for treatment effectiveness. QALYs can be calculated using the EQ-5D [28]. Cost date will be collected in the study.

*Safety assessment* will be performed by monitoring the safety assessment indices, including adverse events, e.g., the frequency of symptomatic intracranial hemorrhage transformation during the hospital stay, vital signs, laboratory tests and Electrocardiograph (ECG) examination.

An adverse event is defined as any untoward medical event experienced in a subject after taking the drug. And an adverse event leading to one or more of the following results is defined as a serious adverse event: 1) death; 2) the risk of immediate death; 3) significant/permanent disabilities or organ damages; 4) deformities and birth defects; 5) hospitalization or extension of hospitalization time; 6) important medical events or intervention measures. The causality between adverse events and the treatments will be judged by the investigators. The severity of adverse events will be evaluated according to the safety assessment indexes. All adverse events that have occurred during the course of the patient’s treatment will have to be reported in the registry timely and accurately. The onset date, the stop date (if the adverse event is considered to have been resolved) and the severity should be entered. Serious adverse events need to be reported to local regulatory authorities, if required. If possible, any symptom or any abnormality should be entered to provide as much medical information as possible.

## Data quality control

### Data quality assurance

Before the initiation of the study, the research staffs undertaking the outcome measurement are trained in use of the data collections tools, assessments and reporting procedures. A data audit team will monitor the data management. During the study, site audit visits by the sponsor and a third-party Contract Research Organization (CRO) will occur on a regular basis to ensure adherence to study documentation, reporting procedures and study protocol. eCRF will be reviewed to ensure the data collected accurately. All data management and supervising procedures must be in accordance to company Standard Operation Procedures (SOPs) for Good Clinical Practice (GCP) Guidelines (Seen in Additional file 1).

### Interim analysis

After 5000 patients are enrolled in the study, we will perform an interim analysis. The results will be reviewed by a CRO, and then submitted to the sponsor and the principal investigator.

## Study sample size

The registry must have a sufficiently large sample size to be representative of the real-world setting. As such, the minimum target number of patients to be included in the registry is 10,000. The choice of sample size is mainly based on the resource available in our study.

## Statistical analysis

Categorical data will be presented as frequencies and percentages. Continuous data will be presented as mean ± standard deviation (SD), and non-normal continuous data will be presented as median with an interquartile range (IQR). The primary efficacy analysis population is intent-to-treat (ITT), defined as all patients who have received at least one drug treatment. The last-observation-carried-forward approach will be used for efficacy assessment in case of missing data. A safety analysis will be performed by analyzing the frequency of adverse events related to the treatment.

## Discussion

The CASTOR is a prospective, multi-center clinical registry with a large sample size. It is designed to investigate the drug treatments for AIS in the real-world settings and to find high cost-utility treatments for AIS.

Considering the study may last for several years, retrospective modifications may be needed when interim data are collected.

A registry could reveal the treatment patterns, such as switching therapies during follow-up and concomitant medications in a real-world setting [29], which are unlikely to be obtained in clinical trials. In a typical clinical trial, the majority of decision-making process is taken by medical doctors and the patients because most medical decisions are strictly regulated by the study protocol. On the contrary, the CASTOR uses an observational design and the treatments will not be specified by the study protocol. On the other hand, biases are common in registry studies. Participants may be less homogeneous in characteristics, some data may be unavailable, and combined treatments can vary widely. Application of propensity score and other statistical means may reduce biases in further studies.

The CASTOR will enroll as many patients as possible, which contribute to finding some high cost-utility treatments of AIS and improving the outcomes of patients with AIS in China.

## Additional file

Additional file 1: Standard Operation Procedures (SOPs) of Chinese Acute Ischemic Stroke Treatment Outcome Registry (CASTOR). (DOCX 109 kb)

## Abbreviations

AIS: Acute ischemic stroke
CFDA: China Food and Drug Administration
CNSR: China National Stroke Registry
CRO: Contract research organization
CT: Computed tomography
ECG: Electrocardiograph
eCRF: Electronic case report form
EQ-5D: EuroQOL five dimensions questionnaire
GCS: Glasgow Coma Scale
IQR: Interquartile range
ITT: Intent-to-treat
MMSE: Mini-mental state examination
MRI: Magnetic resonance imaging
mRS: Modified Rankin Scale
NIHSS: National Institutes of Health Stroke Scale
QALYs: Quality adjusted life years
SD: Standard deviation
TOAST: Trial of Org 10,172 in Acute Stroke Treatment
tPA: Tissue Plasminogen Activator
