# Supplementary material for: Chinese acute ischemic stroke treatment outcome registry (CASTOR): protocol for a prospective registry study on patterns of real-world treatment of acute ischemic stroke in China
Source: BMC Complement Altern Med. 2017 Jul 6;17:357. doi: 10.1186/s12906-017-1863-4 (PMC5501552; doi:10.1186/s12906-017-1863-4)
Supplement: Additional file 1: — Standard Operation Procedures (SOPs) of Chinese Acute Ischemic Stroke Treatment Outcome Registry (CASTOR). (DOCX 109 kb) [file 12906_2017_1863_MOESM1_ESM.docx]

**Clinical protocol**

**Chinese Acute Ischemic Stroke Treatment Outcome Registry, CASTOR**

| **Protocol serial number** | KLK-CBV-2015-001-C |
| --- | --- |
| **Sponsor** | Guangdong Techpool Pharmaceutical Stock Co., Ltd |
| **Principal investigator** | Professor Huang Yining, Peking University First Hospital |
| **Protocol version and date** | March 25, 2015 |
| **Protocol version number** | 1.0 |

| The confidential information contained in this document belongs to Guangdong Techpool Pharmaceutical Stock Co., Ltd. Any third party shall not use the information concerning this document shall not be used without the written permission of Guangdong Techpool Pharmaceutical Stock Co., Ltd., not including the relevant personnel of this study. |
| --- |

Investigator statement

According to GCP regulations, I will carefully fulfill the investigator's duty.

| Name of investigator (signature): | ———————— | Signature of the investigator | ———————— |
| --- | --- | --- | --- |
| Name of study unit | ———————— | Date of signature | ———————— |
|  |  |  |  |
| Name of person-in-charge of Guangdong Techpool Pharmaceutical Stock Co., Ltd. (signature) | ———————— | Signature of person-in-charge of Guangdong Techpool Pharmaceutical Stock Co., Ltd. | ———————— |
| Date of signature | ———————— |  |  |

I have read this protocol, and this project will proceed according to the morality, ethics and scientific principles regulated by Declaration of Helsinki and Chinese GCP. I agree to carry out this clinical study according to this protocol design and regulations.

I will be responsible for making clinically-related medical decisions to guarantee that the subjects can be timely and properly treated in case of the occurrence of adverse reactions during the study. I know the requirements of correctly reporting the serious adverse events. I will truthfully report these events to relevant departments or units in good time in accordance with the requirements.

I guarantee the data will be recorded into the registration platform accurately, completely, timely and legally. I will accept the audit and inspection from the monitors or auditors dispatched from the sponsor as well as the food and drug administration departments, make active responses to the comments or suggestions proposed or the measures adopted by the relevant personnel, and guarantee the quality of clinical study.

I will give necessary guidance and examinations to other participating units according to the requirements, and actively coordinate to cope with the questions encountered by each site during the study to ensure the clinical study conforms to relevant policies and the requirements of this protocol.

As I consent, the sponsor can publicize the study results.

**Table of contents**

Abstract of protocol 4

Study flow diagram 8

Abbreviations 10

1. Brief introduction 11

3 Overview of study design 12

4 Study population 13

1. Inclusion criteria 13

5 Treatment regimen 13

6 Concomitant treatment 14

7 Study procedures 14

Definition and classification of adverse events 23

# Abstract of protocol

| Study title | Registration study on treatment outcome of Chinese acute ischemic cerebral stroke |
| --- | --- |
| **Sponsor** | Guangdong Techpool Pharmaceutical Stock Co., Ltd |
| **Principal Investigator** | Professor Huang Yining, Peking University First Hospital |
| **Study staging** | Non-interventional case registration study |
| **Planned number of study sites and patients** | 10,000 cases |
| **Study schedule** | From March 2015 to March 2017, the expected inclusion time is 18 months |
| Study Background Overview | The stroke is one of the diseases seriously threatening people's health, and has the features of high incidence, high morbidity and high recurrence rate, among which the acute ischemic stroke accounts for about 70% to 80%. The patients will not only experience physical dysfunction, but disorders in mental state, social function and other aspects, which would seriously affect their quality of life, and bring a heavy burden on their families and the society. Therefore, the scientific and rational prevention and proper treatment of stroke is very important.  After the tireless research by scientists and clinical experts, a lot of achievements have been obtained for the prevention and treatment of AIS over the past decade. And in the whole world, there is still no adequately effective drugs or methods for the treatment of AIS, except for the ultra-early thrombolytic treatement and antiplatelet therapy with aspirin.  In China, the situation of the drugs for ALS is particularly complex, and, according to the relevant reports, there are more than 100 types of drugs for the clinical treatment of AIS. There was no data from clinical investigations of large sample size, to guide how to achieve good therapeutic effect, or possess a better cost - effectiveness through the combinations of different types of drugs. |
| Study objective: | Primary study objective:  Analysis of current treatment situation in acute phase of acute ischemic stroke in real-world settings。  Secondary study objectives  (1) Comparison of effects of various drug combinations in treatment of acute ischemic stroke in real-world settings.  (2) Analysis of cost-utility of various drug combinations in treatment of acute ischemic stroke.  (3) Analysis of occurrences of complications and adverse events during treatment in hospital in patients with acute ischemic stroke.  (4) Analysis of effects of TOAST typing on specific therapy regimens in acute phase of acute ischemic stroke. |
| Study design | This was a registered, prospective, multi-center clinical study. The study was planned to included 10, 000 patients with acute ischemic stroke (AIS) and record treatment regimens, treatment outcomes and medical expenses during hospitalization in real clinical practice. This study was to collect patients' data through a electronic data capture system (EDC).  According to the recommendations for treatment and follow-up observations in acute phase in the "Guidelines on diagnosis and treatment of acute ischemic stroke in China 2014 (Draft)", the patients with stroke were planed to receive 5 visits, which occurred at admission (baseline assessment, visit 1), 7 days ± 2 days after medication (visit 2), at discharge (visit 3), 90 days ± 14 days after medication (visit 4, outpatient service or telephone follow-up), and 360 days ± 28 days after medication (visits 5, telephone follow-up), respectively.  In the baseline phase, the investigators should verify the inclusion/exclusion criteria, obtain the patients' informed consent, and try to collect the demographic information, past history, onset and admission conditions, and vital signs of the patients; and collect the following data of clinical examination: ECG, laboratory tests (blood routine, blood glucose, blood lipids, hepatic and renal functions, cardiac enzymes, serum electrolytes, coagulation) and imaging findings, and assess Glasgow Coma Scale, NIHSS score, mRS score and EQ-5D Scale. Seven days ± 2 days after medication, the vital signs of the patients were recorded and the NIHSS scores were accessed again. At discharge, the treatment regimen, vital signs, ECG and above laboratory tests results were recorded during hospitalization, and the Glasgow Coma Scale, NIHSS score, mRS score and EQ-5D scale were accessed, and the medical expenses during hospitalization was also collected. On 90 ± 14 days after medication, the recurrence of stroke, survival conditions and re-admissions were collected; the medical expense until this visits since discharge was recorded; and the mRS score, EQ-5D scale and MMSE scale (only applicable for the patients visiting the hospital) were accessed. On 360 days ± 28 days after medication, another telephone follow-up was given again, to ask the patient's health status, access mRS score, EQ-5D scale, and collect medical expenses.  Only the drug combinations during hospitalization and the occurrences of adverse events during the study period were recorded.  If the patient was discharged on 7 ± 2 days after medication, it should be handled as a discharge note at this time. |
| **Inclusion criteria** | 1. Age≥18 years 2. In conformity with the diagnosis criteria (draft) of China 2014 Guidelines for Diagnosis and Treatment of Ischemic Cerebral Stroke 3. The onset time of thrombolytic patients are within the time window of thrombolysis 4. The onset time of thrombolytic patients are within one week 5. The patients or legal guardians can understand and sign the informed consent form |
| **Exclusion criteria:** | 1. Cranial CT/MRI can display the presence of cerebral hemorrhage 2. The expected survival time of patients less than three months because of complicated serious systemic diseases i 3. the patients cannot provide continuous follow-up information by investigator judgments |
| **Primary efficacy endpoint** | The proportion of patients with good prognosis (mRS=0-2 points) at 90 days after treatment |
| **Secondary efficacy endpoint** | 1. The proportion of patients with poor prognosis (mRS= 3-5 points) and death (mRS=60 points) at 90 days after treatment 2. The changes in National Institutes of Health Stroke Scale (NIHSS) at discharge from baseline 3. The changes in EQ 5 visual analogue scale (EQ-5D, VAS) at discharge from baseline |
| **Safety parameters** | 1. Incidence rate of symptomatic intracranial hemorrhage transformation during the hospital stay 2. Adverse events 3. Vital Signs 4. Laboratory tests (blood routine test, blood glucose, blood lipids, liver and renal functions, myocardial enzymes, serum electrolytes, and coagulation function) 5. ECG examination |
| **Exploratory analysis** | Analysis on the prescriptions of acute specific treatment for different TOAST types  Stroke recurrence rate and incidence rate of post-stroke dementia at 90 days after treatment |
| **Pharmacoeconomic evaluation** | Calculate the cost/utility of the protocol of combinations of different drugs, and ascertain the optimal protocol of hospital treatment of patients with acute cerebral stroke.  As for the costs, only the direct cost is collected, and the indirect cost and implicit cost are not collected. The quality adjusted life years (QALYs) is adopted for the utility analysis as the health outcome parameter. |
| **Statistical methods** | A descriptive statistical analysis is made on the proportion of patients with good prognosis (mRS=0-2 points) and the proportion of patients with poor prognosis (mRS=3-5 points) and death (mRS=6 points) at 90 days after treatment, the corresponding number of cases and the proportion are calculated, and χ^2^ or Fisher's exact test is adopted to compare the inter-group difference. If the inter-group difference is of statistical significance, the Bonferroni method is adopted to adjust the size of test α value, and inter-group pairwise comparison is further made.  The changes in National Institutes of Health Stroke Scale (NIHSS) and EQ 5 visual analogue scale (EQ-5D) at discharge from baseline: if the score difference between before treatment and after treatment is in normal distribution and satisfied homoscedasticity, then analysis of variance is performed. If the statistical test results of the analysis of variance are of statistical significance, statistical analysis is further performed, and the Bonferroni test is carried out for pairwise comparison. Otherwise, the Kruskal Wallis method is adopted to perform statistical test. If the statistical test results of the Kruskal Wallis are of statistical significance, statistical analysis is further performed, and the Wilcoxon rank test is carried out, and the Bonferroni method is adopted to correct P value for pairwise comparison.  The descriptive statistical method is adopted to perform the analysis on all the data. As for the measurement data, number of cases (missing number), mean value, median, standard deviation, first quartile, third quartile, maximum and minimum are described, and the 95% confidence interval of the mean is calculated; as for the enumeration data, the frequency is described. It the hypothesis test needs to be carried out, the corresponding hypothesis test methods are adopted according to the nature of materials and the purpose of the test.  Unless otherwise specified, the hypothesis test adopted by this study is two-sided test with 0.05 as the size of test. P value ≤ 0.05 meant that the difference is of statistical significance.  Based on whether the score difference before and after treatment is in normal distribution, the paired t-test is adopted to make an inter-group comparison on measurement data, or the Wilcoxon signed rank test is used for analysis; χ ^2^ test or Fisher exact probability test is adopted to analyze the enumeration data; the Wilcoxon rank test is adopted to analyze the ranked data. |

#

# Study flow diagram

| **Phase** | **Baseline phase** | **Treatment phase** | | **Follow-up period** | |
| --- | --- | --- | --- | --- | --- |
| Visit | V1 | V2[a](#bookmark) | V3 | V4  (Outpatient or  telephone visit) | V5  (telephone visit) |
| Days | At admission  Day 0 | 7 ± 2 days after administration | At discharge | 90±14 days after administration | 360±28 days after administration |
| Signing informed consent forms | × |  |  |  |  |
| Inclusion / exclusion criteria | × |  |  |  |  |
| Demographic materials and medical insurance type | × |  |  |  |  |
| Visit delay and visits accepted by emergency | × |  |  |  |  |
| Past medical history and risk factors^b^ | × |  |  |  |  |
| Onset and admission^d^ | × |  |  |  |  |
| Glasgow coma score | × |  | × |  |  |
| NIHSS score | × | × | × |  |  |
| mRS score | × |  | × | × | × |
| EQ-5D scale | × |  | × | × | × |
| MMSE scale ^e^ |  |  |  | × |  |
| TOAST classification |  |  | × |  |  |
| Vital Signs^f^ | × | × | × |  |  |
| ECG examination | × |  | × |  |  |
| Laboratory examination^g^ | × |  | × |  |  |
| Imaging examination | × |  |  |  |  |
| Treatment regimen^h^ |  |  | × |  |  |
| Combined medications | × | × | × |  |  |
| Adverse events^i^  Special attention should be paid to the symptomatic intracranial hemorrhagic transformation | At any time | | | | |
| Medical expenses^j^ |  |  | × | × | × |
| Recurrence, survival status and re-admission |  |  |  | × | × |

Note:

1. At visit 2, if the patient is discharged, please directly perform the evaluation related with visit 3.
2. The visit delay, visits accepted by emergency and AIS risk factors are the items that can be evaluated.
3. Past medical history includes: stroke, hypertension, diabetes, dyslipidemia, coronary heart disease, atrial fibrillation, carotid plaques, and history of tumor.
4. Onset and conditions at admission: stroke occurrence time, and main symptoms
5. MMSE scale is only applicable to the patients that receive follow-up visits at 90±14 days after the initiation of administration.
6. The vital signs include body temperature, pulse, respiration and blood pressure. The changes in body temperature 1 day and 24 hours after the onset need to be collected at visit 1.
7. Laboratory tests include: blood routine test, blood glucose, blood lipids, liver and renal functions, myocardial enzymes, serum electrolytes, and coagulation function.
8. The therapeutic regimen refers to AIS specific treatment, and the non-specific treatment needs to be recorded in the combined medication page.
9. The collection of adverse events starts from the signing of informed consent form to 30 days after the initiation of the last administration.
10. The medical expenses refer to the direct costs, including the direct medical costs and and direct non-medical costs. At discharge visit, the direct medical costs are acquired through the expense detailed list when the patient is discharged, and the direct non-medical costs (including the transportation expense, nutrition expense and family accompany expenses) are acquired through the inquiry of the patient or his/her family.

# Abbreviations

| AE | adverse event |
| --- | --- |
| AIS | acute ischemic stroke |
| eCRF | electronic case report form |
| EDC | electronic data capture |
| EQ-5D | EuroQOL five dimensions questionnaire |
| GCP | good clinical practice |
| IEC | independent ethics committee |
| IRB | institutional review board |
| mRS | modified Rankin Scale |
| MMSE | mini mental state examination |
| NIHSS | NIH Stroke Scale |
| QALYs | quality adjusted life years |
| rt-PA | recombinant tissue plasminogen activator |
| SAE | serious adverse event |
| TOAST | Trial of Org 10172 in Acute Stroke Treatment |
| TTO | time trade off |

# Brief introduction

The stroke is one of the diseases seriously threatening people's health, and has the features of high incidence, high morbidity and high recurrence rate , Acute ischemic stroke (AIS) is the commonest cerebral stroke type, and accounts for about 60%~80%[[1]](#bookmark1) of all cerebral stroke. The patients will not only experience physical dysfunction, but disorders in mental state, social function and other aspects, which would seriously affect their quality of life, and bring a heavy burden on their families and the society..The population monitoring materials issued by Statistical Center of China's Ministry of Health, the annual number of newly-emerging cerebral stroke patients nationwide is about 2 million, the annual number of patients that die of cerebrovascular disease is about 1.5 million, and the number of surviving patients is 5 million to 6 million. Among the surviving patients, about 3/4 of them have the loss of labor abilities to varying extents and severely crippled patients account for 40%[[2]](#bookmark2). Therefore, the scientific and reasonable prevention and timely correct treatment of cerebral stroke are of important significance.

Up to now, in the drug treatment for ischemic cerebral stroke, only the ultra-early thrombolytic therapy and the antiplatelet therapy obtain the support of evidence-based medicine proof. For example, the application of rt-PA for intravenous thrombolysis within 3 hours after onset significantly reduces the risk of patient death and severe disability. The European Cooperative Acute Stroke Study (ECASS-III) indicated that it is safe and feasible for the patients to receive intravenous rt-PA treatment at 3 to 4.5 hours after stroke, and it can improve the patient's prognosis [[3]](#bookmark3). The large-scale clinical study on the urokinase thrombolytic therapy in China indicated that, a total of 465 patients with acute ischemic stroke were included into 51 sites, and the results showed that, the adoption of urokinase thrombolytic therapy within 6 hours after onset was safe and effective[[4]](#bookmark4). Based on the above evidence, currently the guidelines in all countries recommend the thrombolytic therapy as the therapeutic measure (class I evidence); however, the actual proportion of patients that receive thrombolysis is very low. In USA, the thrombolytic therapy rate of patients with stroke does not exceed 3.5%[[5]](#bookmark5) while the intravenous thrombolysis rate of patients with ischemic cerebral stroke in China is expected to be 1%~3%[[6]](#bookmark6), so the majority of patients do not benefit from the thrombolytic therapy. As for the patients that receive non-thrombolytic therapy, there is still no effective treatment method supported by evidence-based medicine proofs.

In China, the situation of the drugs for ALS is particularly complex, and, according to the relevant reports, there are more than 100 types of drugs for the clinical treatment of AIS.The study of the China Quality Evaluation of Stroke Care and Treatment (China QUEST) evaluated the treatment of Chinese patients with ischemic cerebral stroke during the acute stage[[7]](#bookmark7), and the results indicated: 1) 75.9% (3632/4783) of the patients with ischemic cerebral stroke received the treatment of intravenous nerve protects, including Edaravone, Ganglioside, Calf cerebroside ignotin injection, Maleate cinepazide injection, Nicholin and other nerve protective agents. 2) The proportion of cerebral stroke that receive intravenous or oral Chinese herbal medicines is up to 83.1%. There are over 100 kinds of prepared Chinese medicines that are domestically used, such as red sange root, rhizome of Sichuan lovage, puerarin and Angongniuhuang injection. Only the patients that receive thrombolytic therapy are mentioned in China 2011 Guidelines for Diagnosis and Treatment of Ischemic Cerebral Stroke [[1]](#bookmark8)about the combined medication, and the use of anti-platelet drugs such as aspirin should be initiated at 24 hours after thrombolysis. Although the nerve protective agents and Chinese herbal medicines are commonly used in clinical practice, there is no high-quality RCT that can prove their efficacy and safety, and its use is not recommended in guidelines.

The results of internal investigation of our company indicated that, the domestic therapeutic regimen for AIS was mainly combined medications; the proportion of the patients that received three-drug regimen and four-drug regimen was relatively high: for example, the proportion of patients that used Chinese herbal medicine + antiplatelet drug + cerebral circulation improving drug was 40%, and the proportion of the patients that used Chinese herbal medicine + antiplatelet drug + cerebral circulation improving drug + nerve protective agents was 87%. One meta analysis[[8]](#bookmark9) included 145 RCT studies, where the data on the efficacy and safety of a total of 13289 Chinese Patients were collected, the main outcome index was NIHSS score and clinical effectiveness rate, and the results indicated that the efficacy of Edaravone + Kininogenase was superior to that of Sodium ozagrel; Edaravone+ Kininogenase, as well as the Sodium ozagrel + Edaravone are the most efficacious therapeutic regimen, and can improve the nerve functional disorder. The systematic evaluation on Chinese herbal medicine ligustrazine in treating acute ischemic cerebral stroke[[9]](#bookmark10) indicates that, the combination of ligustrazine with conventional therapies can better lower the score of nerve functional defects than the conventional therapies (*MD*=2.49, 95%CI: 1.00, 3.98). There is no clear conclusion on whether which combined drug regimen is the most efficacious on the patients who miss the timing of thrombolysis.

The acute ischemic cerebral stroke is the main cause that leads to the long-term disenabling, thus bring severe economic burdens[[10]](#bookmark11) to society and patient's family. Therefore, the performing of pharmacoeconomic evaluation on the different therapeutic regimens during the acute phase of cerebral stroke and the seeking of one or more kinds of drug therapeutic regimens with a high price/performance ratio which are suitable to the domestic conditions are of a higher social value. In the field of cerebral stroke treatment, currently there is rare prospective pharmacoeconomic evaluation domestically. Unlike the domestic cerebral stroke study fields which often adopt the cost effectiveness analysis (CEA)[[11]](#bookmark12), this study utilizes the cost utility analysis (CUA) for economic evaluation, the quality adjusted life years (QALYs) serves as the health outcome index, and the advantage of this method lies the measurement of life quality and the improvement of life-span (longevity) [[12]](#bookmark13). Considering the original intention of using this study as the registration study as well as the difficulty in expense collection, this study only collects the direct costs during the patient's hospitalization, and does not collect the indirect cost and implicit cost.

To sum up, this objective of this study is to analyze status quo of different drug combinations in the real world, evaluate the efficacy of different drug combination regimens in treating acute ischemic cerebral stroke, and perform cost-utility analysis. It aims to provide reference for a wide range of clinicians when selecting medications to treat acute ischemic cerebral stroke.

**2 Study objective**

Primary study objective:

Analysis of current treatment situation in acute phase of acute ischemic stroke in real-world settings。

Secondary study objectives

(1) Comparison of effects of various drug combinations in treatment of acute ischemic stroke in real-world settings.

(2) Analysis of cost-utility of various drug combinations in treatment of acute ischemic stroke.

(3) Analysis of occurrences of complications and adverse events during treatment in hospital in patients with acute ischemic stroke.

(4) Analysis of effects of TOAST typing on specific therapy regimens in acute phase of acute ischemic stroke.

# 3 Overview of study design

This was a registered, prospective, multi-center clinical study. The study was planned to included 10, 000 patients with acute ischemic stroke (AIS) and record treatment regimens, treatment outcomes and medical expenses during hospitalization in real clinical practice. This study was to collect patients' data through a electronic data capture system (EDC).

According to the recommendations for treatment and follow-up observations in acute phase in the "Guidelines on diagnosis and treatment of acute ischemic stroke in China 2014 (Draft)", the patients with stroke were planed to receive 5 visits, which occurred at admission (baseline assessment, visit 1), 7 days ± 2 days after medication (visit 2), at discharge (visit 3), 90 days ± 14 days after medication (visit 4, outpatient service or telephone follow-up), and 360 days ± 28 days after medication (visits 5, telephone follow-up), respectively. As for the specifics, refer to [Study Flow Chart](#bookmark14).

In the baseline phase, the investigators should verify the inclusion/exclusion criteria, obtain the patients' informed consent, and try to collect the demographic information, past history, onset and admission conditions, and vital signs of the patients; and collect the following data of clinical examination: ECG, laboratory tests (blood routine, blood glucose, blood lipids, hepatic and renal functions, cardiac enzymes, serum electrolytes, coagulation) and imaging findings, and assess Glasgow Coma Scale, NIHSS score, mRS score and EQ-5D Scale. Seven days ± 2 days after medication, the vital signs of the patients were recorded and the NIHSS scores were accessed again. At discharge, the treatment regimen, vital signs, ECG and above laboratory tests results were recorded during hospitalization, and the Glasgow Coma Scale, NIHSS score, mRS score and EQ-5D scale were accessed, and the medical expenses during hospitalization was also collected. On 90 ± 14 days after medication, the recurrence of stroke, survival conditions and re-admissions were collected; the medical expense until this visits since discharge was recorded; and the mRS score, EQ-5D scale and MMSE scale (only applicable for the patients visiting the hospital) were accessed. On 360 days ± 28 days after medication, another telephone follow-up was given again, to ask the patient's health status, access mRS score, EQ-5D scale, and collect medical expenses.

Only the drug combinations during hospitalization and the occurrences of adverse events during the study period were recorded.

If the patient was discharged on 7 ± 2 days after medication, it should be handled as a discharge note at this time.

As the database can last for several years, whenever there is a need to supplement or modify the data, a retrospective modification / amendment can be carried out at any time. According to the inclusion, data tendency and management factors, it is possible that the duration may be longer; therefore, the patients who are preliminarily included and added during the late stage may be followed up after 3 months.

Techpool can terminate this study at any time due to any reason. The physicians participating in the disease registration study is responsible for notifying IRBs and/or ECs about the premature termination of the study.

If necessary, the disease evaluation can be evaluated more frequently. In this study, any valuable information collected at follow-up visit is recorded.

# 4 Study population

The age of the population included into this study is not less than 18 Y, and the diagnosis at admission is acute ischemic cerebral stroke, male or female.

## Inclusion criteria

The patients who satisfy all of the following criteria can be included into this study:

(1) Age≥18 years

(2) In conformity with the diagnosis criteria (draft) of China 2014 Guidelines for Diagnosis and Treatment of Ischemic Cerebral Stroke

(3) The onset time of thrombolytic patients are within the time window of thrombolysis

(4) The onset time of thrombolytic patients are within one week

(5) The patients or legal guardians can understand and sign the informed consent form

**4.2 Exclusion criteria:**

(1) Cranial CT/MRI can display the presence of cerebral hemorrhage

(2) The expected survival time of patients less than three months because of complicated serious systemic diseases

(3) The patients cannot provide continuous follow-up information by investigator judgments

# 5 Treatment regimen

In the study, the doctors can select the specific treatment regimen for cerebral stroke according to the patient's clinical medical history and characteristics of symptoms as well as the China 2011 Guidelines for Diagnosis and Treatment of Ischemic Cerebral Stroke. This study has no limitations on the selection, dose and treatment course of the treatment drugs.

According toChinese Guideline for Diagnosis and Management of Acute Ischemic Stroke2010, The specific treatment on acute ischemic cerebral stroke refers to the intervention with a certain link in the pathophysiological mechanism of ischemic injuries. Examples are given to the following common drugs for illustration:

1. Intravenous thrombolysis drug: rt-PA and urokinase.
2. Anti-platelet drugs: aspirin and Clopidogrel
3. Anticoagulants: including unfractionated heparin, low molecular heparin, ateroid, oral decoagulants and thrombin inhibitor.
4. Volume expansion drug: hydroxyethyl starch, and low molecular dextran.
5. Nerve protective agents: Edaravone, nicholin and ganglioside.
6. Cerebral blood flow perfusion improving drugs: butylphthalide, human urine kininogenase (Urinary Kallidinogenase for Injection).
7. Chinese herbal medicines: Shuxuetong injection, and Danhong injection.

# 6 Concomitant treatment

Combined medications: any other drug given during the inpatient treatment except the specific treatment for acute ischemic cerebral stroke.

The cerebral infarction non-specific treatment drugs include dehydrants, antihypertensive drugs, antihyperlipemic drugs, hypoglycemic drugs, and anti-infective drugs etc.

When the patients are included into the study (namely first visit), all accompanying diseases and combined medications must be recorded in detail. At the discharge visit, it is necessary to record any change in the combined medications in detail.

For each kind of combined medications, the following information should be collected: generic name, dose, initial date, ending date or continuous use, and indications.

# 7 Study procedures

**7.1 Baseline phase**

**Visit1 (At admission Day 0)**

- Signing informed consent forms
- Verify the inclusion / exclusion criteria
- Demographic materials (Sex, Age) and medical insurance type
- Visit delay and visits accepted by emergency*

The items with a "*" can be evaluated.

- Past medical history (stroke, hypertension, diabetes, dyslipidemia, coronary heart disease, atrial fibrillation, carotid plaques, and history of tumor), the history of medications during the recent three days, and risk factors*

The items with a "*" can be evaluated.

- Onset and admissiond (Stroke occurrence time, and main symptoms)
- Imaging examination (Cranial CT/MRI and vascular examination)

If possible, the researchers are expected to burn the disks to store the patient's imaging materials (cranial CT / MRI imaging)

- Vital Signs (Body temperature, pulse, respiration and blood pressure)

The changes in body temperature 1 day and 24 hours after the onset need to be collected.

- NIHSS score
- mRS score
- EQ-5D scale
- ECG examination
- Laboratory tests (blood routine, blood glucose, blood lipids, hepatic and renal functions, cardiac enzymes, serum electrolytes, coagulation)
- Concomitant treatment
- Adverse events

**7.2 Treatment phase**

**Visit 2 (7 ± 2 days after administration)**

- Vital Signs (Body temperature, pulse, respiration and blood pressure)
- NIHSS score
- Concomitant treatment
- Adverse events

**Visit 3 (At discharge)**

- Vital Signs (Body temperature, pulse, respiration and blood pressure)
- Glasgow coma score
- TOAST classification
- NIHSS score
- mRS score
- EQ-5D scale
- ECG examination
- Laboratory tests (blood routine, blood glucose, blood lipids, hepatic and renal functions, cardiac enzymes, serum electrolytes, coagulation)
- Therapeutic regimen (namely the specific treatment of acute ischemic cerebral stroke)

The name (generic name), dose, administration method, initial medication time and medication duration of the treatment drug need to be recorded.

- Collect medical expenses

The direct medical costs are acquired through the expense detailed list when the patient is discharged, and the direct non-medical costs (including the transportation expense, nutrition expense and family accompany expenses) are acquired through the inquiry of the patient or his/her family.

The contents of the collected direct medical costs include:

1. Total inpatient expenses (including the self-paid part)
2. Category of comprehensive medical service: including general medical service expense, general treatment and procedure expense, nursing expense and other expenses
3. Category of diagnosis: including pathological diagnosis expense, laboratory diagnosis expense, imaging diagnosis expense, and clinical diagnosis expense
4. Category of treatment: including expense of non-surgical item and surgical treatment expense
5. Rehabilitation expense
6. TCM therapy expense
7. Western medicine expense
8. Category of Chinese medicines: including expense of prepared Chinese medicine and expense of Chinese herbal medicine
9. Category of blood and blood products: including expense of blood, expense of protein-like products, expense of globulin-like products, expense of blood coagulation factor like products, and expense of cytokine like products.
10. Category of consumables: including the expense of disposable medical materials for examination, the expense of disposable materials for treatment, and the expense of disposable materials for surgery
11. Other expense

- Concomitant treatment

The non-specific treatment drugs for acute cerebral infarction (dehydrants, antihypertensive drugs, antihyperlipemic drugs, hypoglycemic drugs, and anti-infective drugs etc.), supporting therapy, and drugs for the treatment of accompanying diseases.

- Adverse events

**7.3 Follow-up period**

**Visit 4(After administration 90 ± 14 days, Outpatient or telephone visit)**

- mRS score
- MMSE scale (Only applicable to the patients that receive follow-up visits at hospitals)
- EQ-5D scale
- Collect medical expenses

The patient's medical expenses from discharge to visit 4 are collected (AIS related medical expense, not including the medical expenses for other diseases), including direct medical cost (including the dug expense, examination expense, laboratory test expense, diagnosis and treatment expense, nursing expense and bed expense due to outpatient service / emergency service / hospitalization) as well as direct non-medical cost (including transportation expense, nutrition expense, family accompanying expense and the expense of auxiliary devices). The above expenses are obtained through the inquiry from the patient and (or) family. Where the expense list cannot be provided, the estimate value verbally given prevails.

- Recurrence

The patient should be inquired about the recurrence, and the date of recurrence and whether the recurrence leads to inpatient treatment should be recorded.

Note: whether the disease recurs is based on the diagnosis of the medical institution verbally told by the patient or his/her cerebral infarction.

The stroke recurrence is defined as: 1) in conformity with the diagnosis criteria of ischemic cerebral stroke; 2) the history of past stroke with clear symptom; 3) infarction foci at different periods verified by cranial CT/MRI[[13]](#bookmark15).

- Survival status

The patients should be inquired about the survival status, and as for the dead patients, it is necessary to record the date and reason of death.

- Re-admission

The patient should be asked whether he/she is re-admitted as well as the frequency and reasons of admission.

**Visit 5 (360 ± 28 days after administration, telephone visit)**

- mRS score
- EQ-5D scale
- Collect medical expenses

The patient's medical expenses from visit 4 to visit 5 should be recorded. (AIS related medical expense, not including the medical expenses for other diseases), including direct medical cost (including the dug expense, examination expense, laboratory test expense, diagnosis and treatment expense, nursing expense and bed expense due to outpatient service / emergency service / hospitalization) as well as direct non-medical cost (including transportation expense, nutrition expense, family accompanying expense and the expense of auxiliary devices). The above expenses are obtained through the inquiry from the patient and (or) family. Where the expense list cannot be provided, the estimate value verbally given prevails.

• Recurrence

The patient should be inquired about the recurrence, and the date of recurrence and whether the recurrence leads to inpatient treatment should be recorded.

Note: whether the disease recurs is based on the diagnosis of the medical institution verbally told by the patient or his/her cerebral infarction.

The stroke recurrence is defined as: 1) in conformity with the diagnosis criteria of ischemic cerebral stroke; 2) the history of past stroke with clear symptom; 3) infarction foci at different periods verified by cranial CT/MRI[13].

• Survival status

The patients should be inquired about the survival status, and as for the dead patients, it is necessary to record the date and reason of death.

• Re-admission

The patient should be asked whether he/she is re-admitted as well as the frequency and reasons of admission

- Study completed / ending page

**8 Study Evaluation**

**8.1 Efficacy evaluation**

Primary efficacy endpoint: The proportion of patients with good prognosis (mRS=0-2 points) at 90 days after treatment

Secondary efficacy endpoint:

(1) The proportion of patients with poor prognosis (mRS= 3-5 points) and death (mRS=60 points) at 90 days after treatment

(2) The changes in National Institutes of Health Stroke Scale (NIHSS) at discharge from baseline

(3) The changes in EQ 5 visual analogue scale (EQ-5D, VAS) at discharge from baseline

**8.2 Safety evaluation**

The safety indexes in this study include:

1. Incidence rate of symptomatic intracranial hemorrhage transformation during the hospital stay
2. Adverse events
3. Vital Signs
4. Laboratory tests (blood routine test, blood glucose, blood lipids, liver and renal functions, myocardial enzymes, serum electrolytes, and coagulation function)
5. ECG examination

**8.3 Pharmacoeconomic evaluation**

Calculate the cost/utility of the protocol of combinations of different drugs, and ascertain the optimal protocol of hospital treatment of patients with acute cerebral stroke. As for the costs, only the direct cost is collected, and the indirect cost and implicit cost are not collected. The direct costs in the study include:

- Direct medical cost (including the dug expense, examination expense, laboratory test expense, diagnosis and treatment expense, nursing expense and bed expense)

Discharge visit (Visit 3): Collect the direct medical cost according to the fees list of patients on discharge.

Visit 4 and visit 5: collect the direct medical costs by inquiring the patients or their families.

- Direct non-medical cost (including transportation expense, nutrition expense, family accompanying expense)

Directly collect the transportation costs and the nutrition costing by inquiring the patients or their families, and collect the nursing fees of the families by inquiring the families of the days/hours of downtime and calculating the fees through multiplying the days/hours by the local average wage.

In the utility analysis, quality-adjusted life years (QALYs) are used as the index of health outcome. The cost unit is Renminbi (RMB).

The QALY values are calculated through the utility value acquired by EQ-ED (evaluated on day 0 before treatment, at discharge,on 90 ± 14 days after treatment, 360 ± 28 days after treatment), and the pathway of the quality of life can be acquired by connecting the utility values on day 0, at discharge, on 90 ± 14 days after medication. With the time required for improving the quality of life as weights, we will get the QALY values. See the reference for the calculation method. [[14]](#bookmark16)

The EuroQol-5 Dimensions (EQ-5D) consists of two parts: the questionnaire and the conversion table of utility values. The questionnaires results can be used to describe the health status of the population and access to the EQ-VAS scores, and the EQ-5D index score can be further acquired by using the the time trade off (TTO) conversion table of utility values. Given that there is no suitable TTO conversion table of utility values in Chinese, the conversion table of utility values for Japanese is used to acquire the EQ-5D index scores. The following is the TTO conversion table of utility values in Japanese [[15]](#bookmark17):

| **Dimensions of EQ-5D** | **Level** | **Index** |
| --- | --- | --- |
| Mobility | 1 | 0.000 |
|  | 2 | 0.075 |
|  | 3 | 0.418 |
| Self Care | 1 | 0.000 |
|  | 2 | 0.054 |
|  | 3 | 0.102 |
| Usual Activities | 1 | 0.000 |
|  | 2 | 0.044 |
|  | 3 | 0.133 |
| Pain or discomfort | 1 | 0.000 |
|  | 2 | 0.080 |
|  | 3 | 0.194 |
| Anxiety or depression | 1 | 0.000 |
|  | 2 | 0.063 |
|  | 3 | 0.112 |
| Constant | 0.152 | |

**8.4 Exploratory Analysis**

(1) Analysis of the effects of TOAST typing on specific therapy prescriptions in acute phase of AIS.

(2) Analysis of the relapse rate and the incidence of stroke dementia in three months after treatment.

**9 Patients complete / Withdraw from the study**

**9.1 Completion**

If the patient can provide all assessment data within the 90 days ± 14 days completely, this patient will be regarded as a completion case.

**9.2 Withdraw from the study**

If the patient experiences any of the followings, the patient should withdraw from this study:

- Wrong inclusion of the patient
- Withdrawal of informed consent
- When any other conditions occur, in the investigator's opinions, terminating this trial is to the subject’s best interests
- Lost to follow-up
- Other

The reason and date of withdrawal should be noted in the electronic Case Report Form (eCRF). Upon terminating the study, a last end-point evaluation should be done, except for lost of follow-up.

**10 Statistical methods**

The descriptive statistical method is adopted to perform the analysis on all the data. As for the measurement data, number of cases (missing number), mean value, median, standard deviation, first quartile, third quartile, maximum and minimum are described, and calculate the 95% confidence interval of the rates. Based on whether the score difference before and after treatment is in normal distribution, the paired t-test is adopted to make an inter-group comparison on measurement data, or the Wilcoxon signed rank test is used for analysis; χ ^2^ test or Fisher exact probability test is adopted to analyze the enumeration data; the Wilcoxon rank test is adopted to analyze the ranked data.

If needed, the missing data of visits in efficacy analysis will be filled in using last observation carried forward (LOCF) method.

**10.1 Determination of sample size**

For a more comprehensive collection of the patient information on the use of various drug combinations, this study protocol is planned to include 10,000 patients with acute stroke.

**10.2 Analysis Data Sets**

All subjects who receive at least 1 dose of investigational drug and have at least 1 efficacy evaluation data after baseline are included in FAS, according to the basic principle of intention-to-treat (ITT) analysis.

**10.3 Analysis of efficacy**

FAS analysis is to be conducted for the efficacy indexes.

A descriptive statistical analysis is made on the proportion of patients with good prognosis (mRS=0-2 points) and the proportion of patients with poor prognosis (mRS=3-5 points) and death (mRS=6 points) at 90 days after treatment, the corresponding number of cases and the proportion are calculated, and χ^2^ or Fisher's exact test is adopted to compare the inter-group difference. If the inter-group difference is of statistical significance, the Bonferroni method is adopted to adjust the size of test α value, and inter-group pairwise comparison is further made.

The changes in National Institutes of Health Stroke Scale (NIHSS) and EQ 5 visual analogue scale (EQ-5D) at discharge from baseline: if the score difference between before treatment and after treatment is in normal distribution and satisfied homoscedasticity, then analysis of variance is performed. If the statistical test results of the analysis of variance are of statistical significance, statistical analysis is further performed, and the Bonferroni test is carried out for pairwise comparison. Otherwise, the Kruskal Wallis method is adopted to perform statistical test. If the statistical test results of the Kruskal Wallis are of statistical significance, statistical analysis is further performed, and the Wilcoxon rank test is carried out, and the Bonferroni method is adopted to correct P value for pairwise comparison.

**10.4 Safety Analyses**

Descriptive statistics are mainly used for safety analysis. The incidences of symptomatic intracranial hemorrhage transformation between the groups are described statistically and compared by using the test of χ^2^ or the exact test of Fisher.

According to the relevant regulations of the state, the SAE incidence of the relevant product of the sponsor shall be described.

The vital signs and the laboratory test mesasurements shall be statistically described and compared by using Student's paired t-test and the Wilcoxon signed rank test befoer and after the baseline. The changes of laboratory test results and the ECC indexes are described with cross tabulations before and after the assessment.

**10.5 Pharmacoeconomic analysis**

The cost - utility evaluation methods are used for the pharmacoeconomic analysis of the drug and the sensitivity analysis for the cost and utility indexes.

**10.6 Exploratory Analysis**

For the recurrence rate of stroke and the incidence of dementia after stroke, the case numbers and their percentage shall be described and compared between the groups by using the test of χ2 or the exact test of Fisher. If the difference between groups is statistically significant, the Bonferroni method shall be used to adjust the significance level of α value, and the comparison between the two groups will be conducted further.

The prescription of specific treatment in the acute phase of different TOAST type will be described by listing.

**10.7 Interim analysis**

After starting, an interim analysis will be conduced when a certain number of patients were enrolled; when the data is collected for the data set and entered into the database, the first interim analysis of management is to be conducted, to check whether the required "core" data collection is allowed to perform a meaningfully preliminary data analysis. Then the subsequent interim data analysis is regularly scheduled based on the research progress and the object is the whole database content.

With the approval of Guangdong Techpool Biopharmaceutical Co., Ltd, and the Scientific Recommendation Commission, the data collected in this study may be merged with data from other areas for investigation and analysis in the future. In accordance with applicable laws and regulations, the patients information in the study shall be confidentially managed.

The detailed description of the specific content of of the interim analysis will be provided in the statistical analysis plan (SAP).

The results of the interim analysis will be submitted to the sponsor and the principal investigators by the the co-sponsor (CRO) in the style of statistical analysis report (SAR) and slides.

**11 Reports of Adverse events/ Serious adverse events (AE/SAE)**

In a clinical study, it is extremely important for protecting the patients, investigators and the sponsor by timely, accurately and completely reporting and analyzing the safety information from the clinical study, meanwhile, the sponsor, investigators and the monitor shall fulfill their respective responsibilities in accordance with the Good Clinical Practice (JL No. 3), Adverse Drug Event Reporting and Monitoring Management Methods (WSBL No. 81) and Drug Registration Management Methods (JL No. 28).

Note: this study is initiated by the sponsor (Guangdong Techpool Biopharmaceutical Co., Ltd); in accordance with the sponsor's SOP "Pharmacovigilance Policy", the investigators should submitted the adverse events/serious adverse events/pregnancy events in the patients using the regiterred drug from the sponsor () to Guangdong Techpool according to the protocol and clinical judgement.

**11.1 Relevant Definitions**

Definition and classification of adverse events

**Adverse Event (AE)**

An adverse event (AE) refers to any untoward medical event experienced by patients or subjects after taking the drug, but the event does not necessarily have a causal relationship with the treatment. Therefore, an adverse event (AE) can be any untoward and unexpected signs (including abnormal investigation findings), symptoms or disease that are temporally related to the used (investigational) drug regardless of whether the event is related to the drug.

**Serious adverse events (SAE)**

Serious adverse event (SAE) refers to any adverse medical event that conforms to one or more of the following criteria under any dose:

• Fatal (note: death is the consequence, not the event)

• Life-threatening (Note: "life-threatening" means the patient has the risk of immediate death when the event occurs, and it does not refer to the hypothesis that death will be induced if the event is more serious.

• Causing significant or permanent human wounds and disabilities or organ functional impairment

• Causing deformities and birth defects

• Causing hospitalization or extension of hospitalization time

• Important medical events or needing intervention measures (the above-mentioned circumstances may arise in case of no treatment)

**Causality of events with the investigational drug**

the causality of events with the investigational drug shall be judged by the investigator according to the following criteria:

Definitely related (must meet five of the following criteria)

This category refers to the adverse event that can be determined to be related with the investigational drug. In case of conformity with the following criteria, it can be considered that one adverse event is "definitely related":

1. The occurrence of adverse events and drug application have a reasonable time-dependency.

2. Patient's known disease state, environmental or toxic factors or patient's use of other treatment cannot reasonably explain the adverse events.

3. After dose discontinuation or reduction, the adverse events will disappear or become relieved. (But there are some important exceptions; as for some obviously drug-related adverse reactions, they not disappear even after the drug withdrawal; such as: (1) bone marrow suppression, (2) tardive dyskinesia.)

4. Adverse events are consistent with the suspected drug reaction mode.

5. Adverse events re-occur in case of the re-use.

6. External factors can be ruled out.

**Probably related (the first three items must be complied with)**

This category refers to the adverse event that can be highly determined to be related with the investigational drug. In case of conformity with the following criteria, it can be considered that one adverse event is "probably related":

1. The occurrence of adverse events and drug application have a reasonable time-dependency.

2. Patient's known disease state, environmental or toxic factors or patient's use of other treatment cannot reasonably explain the adverse events.

3. After dose discontinuation or reduction, the adverse events will disappear or become relieved. (But there are some important exceptions; as for some obviously drug-related adverse reactions, they not disappear even after the drug withdrawal; such as: (1) bone marrow suppression, (2) tardive dyskinesia.

4. Adverse events are consistent with the suspected drug reaction mode or not the known adverse drug reactions.

5. Adverse events re-occur in case of the re-use.

• Possibly related (the first two items must be complied with)

This category refers to the adverse event that is not probably related with the administration of the investigational drug but cannot definitely rule out the relevancy. In case of conformity with the following criteria, it can be considered that one adverse event is "possibly related":

1. The occurrence of adverse events and drug application have a reasonable time-dependency.

2. Adverse reactions may be caused by patient's disease state, the environmental or toxic factors or other combined therapies used by the patient.

3. Adverse events are consistent with the suspected drug reaction mode.

• Unlikely related (the first two items must be complied with)

Typically, this category applies to adverse events with meet the following criteria:

1. The occurrence of adverse events and drug application do not have a reasonable time-dependency.

2. Adverse reactions are significantly caused by patient's disease state, the environmental or toxic factors or other combined therapies used by the patient.

3. Adverse events are inconsistent with the suspected drug reaction mode.

4. The adverse events do not re-occur or no exacerbation occurs in case of re-medication.

• Unrelated

This category refers to the adverse events that are clearly and explicitly determined to be only induced by external factors (diseases and environment etc.) and do not conform to the criteria to judge the drug relevancy under the items of "unlikely related", "possibly related" or "probably related".

| Tables for determination of relevancy between adverse events and investigational drug | | | | | |
| --- | --- | --- | --- | --- | --- |
|  | Definitely related | Probably related | Possibly related | Unlikely related | unrelated |
| Obviously induced by external factors | - | - | - | - | + |
| Have a reasonable time-dependency with the drug application. | + | + | + | - | - |
| Can be induced by factors such as patient's diseases | - | - | + | + | + |
| Consistent with the known reaction mode of the suspected drug | + | +/- | + | - | - |
| Relief or disappearance of dose reduction or drug withdrawal | + | + | - | - | - |
| Re-occurrence of re-medication | + | + | - | - | - |

In order to maximally lower the medication population risk and conform to the requirements of low supervision, the sponsor will handle the event relevancy according to the following modes:

• As for the "unrelated", it is considered to be "unrelated"

• As for the "definitely related", "unlikely related", "possibly related" or "probably related", it is considered to be "related"

• As for "unjudgeable" or "the investigator does not provide relevance judgment", it is considered to be "related".

**Severity criteria**

The severity should be evaluated according to the following classification and description:

- Mild: the symptoms can be aware but tolerated; slight discomfort may occurs; daily activities are not affected;;
- Moderate: the discomforts may affact daily activities.
- Severe: very anguish induce significantly functional loss and lost of self-care ability; is not able to perform the daily activities;

The investigators should not evaluate the severity of events according to the clinical practice instead of the subjects' direct sensations (in case of abnormal laboratory test results).

**11.2 Reporting procedures**

**Adverse Events Reporting regulations:**

Time limit: within 24 hours

In the clinical study (from the time of signed ICF through 30 days after last dose of study medication), the subject should be given of appropriate therapeutic measures in case of any adverse event, no matter what kind of treatment received. The investigators or the authorized personnel must report them to the food and drug supervisory departments of the corresponding provinces, autonomous regions and municipalities directly under the Central Government within the time limit specified by the regulations based on the Adverse Drug Reaction Reporting and Monitoring Management Methods (WSBL No. 81) in case that the investigators judge that this event is not anticipatory. In accordance with the sponsor's SOP "Pharmacovigilance Policy", for the adverse events in the patients using the registered drug from the sponsor (Guangdong Techpool Pharmaceutical Stock Co., Ltd), the investigators should fill in Guangdong Techpool "Adverse Drug Event Report form", and submit the report form in written form to Guangdong Techpool within 24 hours after awareness:

| Guangdong Techpool Pharmaceutical Stock Co., Ltd. - Pharmacovigilance contact mode | |
| --- | --- |
| Hoteline | 400-688-9935 |
| E-mail | DSO-TP@techpool.com.cn |
| Address | Room 3606, Libao Square, No. 222, Middle Huaihai Road, Luwan District, Shanghai |
| Post code | 200012 |

**Regulations on emergency reporting of serious adverse events**

Time limit: within 24 hours

In the clinical study (from the time of signed ICF through 30 days after last dose of study medication), the subject should be given of appropriate therapeutic measures in case of any serious adverse event, no matter what kind of treatment received. The investigators or the authorized personnel must report the reports to the food and drug supervisory departments, other sites and ethic committees of the corresponding provinces, autonomous regions and municipalities directly under the Central Government within the time limit specified by the regulations according to Good Clinical Practice (JL No. 3), Adverse Drug Event Reporting and Monitoring Management Methods (WSBL No. 81) and Drug Registration Management Methods (JL No. 28).

Meanwhile, for the serious adverse events in the patients using the registered drug from the sponsor (Guangdong Techpool Pharmaceutical Stock Co., Ltd), the investigators should fill in Guangdong Techpool "Adverse Drug Event Report form", and submit the report form in written form to Guangdong Techpool within 24 hours after awareness:

| Reporting institution | 24-hour reporting telephone | E-mail |
| --- | --- | --- |
| Institutional review boards | 010-83572075  010-83572507 | kyc@bjmu.edu.cn |
| Guangdong Techpool Pharmaceutical Stock Co., Ltd. | 400-688-9935 | DSO-TP@techpool.com.cn |

For all serious adverse events, regardless of whether the subject has discontinued or completed the study treatment, the investigators need to continue to monitor and report on the SAE until it is healed, stabilized or returns to the baseline status.

Any follow-up information of the serious adverse events should be reported in the written form within 24 hours according to the above procedures.

**Pregnancy reporting regulations:**

Time limit: within 24 hours

If the subject/partner becomes pregnant during the study, the investigators or the authorized personnel should fill in Guangdong Techpool " Pregnancy Event Report form"i, and report the pergenancy status in written form to Guangdong Techpool within 24 hours after awareness.

The investigators should propose advice to the subject, and inform the subject of the dangers of continuation of the pregnancy and the possible effects on the fetus. And the monitoring on the subject should be continued until one month after the end of pregnancy. The information related to the pregnancy outcomes (including newborns) must be submitted to the sponsor.

| Guangdong Techpool Pharmaceutical Stock Co., Ltd. - Pharmacovigilance contact mode | |
| --- | --- |
| Hoteline | 400-688-9935 |
| E-mail | DSO-TP@techpool.com.cn |
| Address | Room 3606, Libao Square, No. 222, Middle Huaihai Road, Luwan District, Shanghai |
| Post code | 200012 |

**12. Study-specific supplies**

The following trial supplies will be provided to the investigators:

• Protocol

• Case report form

• Informed consent forms

**13 Ethical requirements**

**13.1 Responsibilities of the investigators**

The investigators are responsible for ensuring that the clinical trial is executed according to the requirements of the protocol, existing ICH Good Clinical Practice (GCP) and SFDA-related regulations.

ICH-GCP is an internationally-acknowledged ethical and scientific quality standard related to the design, implementation, recording and reporting with human body as the subject objects. The studies in conformity with this standard is considered to satisfy the principles clarified in the Declaration of Helsinki in terms of protecting subject's rights, safety and benefits, and it is trust-worthy in terms of the quality of the study data.

**13.2 Independent Ethics Committee (IEC)**

Before the initiation of this study, the investigators must provide the following documents:

• Final draft of protocol (and supplement);

• Sponsor-approved informed consent (and other written materials provided to the subjects);

(e.g.: Study participating cards and subject diary cards);

- Materials assisting the enrollment of the subject;
- Materials concerning the trial-related injury compensation or the subjects’ participation into the trial;
- Resume or equivalent information of the principal investigators (unless not required by IEC);
- Information concerning the name of the sponsor, the research funds, potential conflicts of interest and the impact on the motivation of a subject participating in the study;
- Other documents required by the IEC.

Only under the condition that IEC/IRB completely approves the study protocol, informed consent form, subjects’ inclusion materials, and measures to compensate the subjects and that the sponsor receives the copies of the approval documents from the IEC, the trial can be initiated. The approval documents should be noted of the study title (number), name of the study document (including version number) and the approval date.

**The investigators may present the following documents for the review and approval of IEC due to some reasons at the proper time point.**

• Supplementation of the protocol

• Modification of informed consent and other written materials provided to the subjects;

• Modified materials concerning the trial-related injury compensation or the subject's participation into the trial;

- New information that may have a negative influence on the safety of the subjects and study implementation;
- Reports on dead subjects under the watch of the investigators;
- Reports on dead subjects;
- Notice on the replacement of the principal investigator in a site;
- Other requirements of IEC/IRB.

If the amendment of the protoco may increase the risk of the subjects, the protocol amendment and the informed consent forms after the corresponding modifications must be rapidly presented to the IEC/IRB for review, and the execution can be performed after approval is obtained.

The IEC should review and approve the study at least once a year and the review comment should be recorded in wirtten.

At the end of the study, the investigators must notify that the IEC trial has ended.

**13.3 Informed consent**

Each subject (or his/her legal representative) must give the written consent after a full understanding of the nature of the study, and this written consent must be signed before the implementation of any trial-related procedures (attach signature and date). The informed consent form must be approved by both sponsor and IEC/IRB, and the language readable and understandable to the subjects should be used. The informed consent form should conform to the Declaration of Helsinki, the existing GCP guidelines, the corresponding regulations, and the regulations provided by the sponsor.

Before the inclusion of the potential subjects, the investigators or their authorized personnel should explain to them the purpose, methods, possible benefits, potential risks and any discomfort that may arise. The subjects should be informed that their participation into the trial should be voluntary and they may withdraw at any time. Whether to choose to participate in the study will not have any influence on his (or her) disease treatment. If the subjects reject the participation in study, they can still choose other treatments, and this rejection will not have an influence on the subsequent treatments. Finally, the subjects should understand that, the investigators will keep their identity records which may be used for the long-term follow-ups if necessary, and the records may be reviewed by pharmaceutical administration department personnel and the personnel of sponsor. within the allowable range of relevant laws and regulations. The subjects' right of privacy will be protected. The signing of the informed consent form means the subjects have authorized the above behaviors.

The subjects (and their legal representatives) should have sufficient time to read the informed consent forms and raise questions. After explanations are given to the investigators and the subjects are included, the subjects (or their legal representatives) should sign their names and affix the dates on the informed consent forms and make records. After signing the informed consent form, the subjects should obtain the copies of the informed consent forms.

If the subjects (and legal representatives) are unable to read and write, there should be equitable witnesses participating in the entire process of informed consent (including reading and explaining all written information) and should sign the names and affix the date after the oral consent given by the subjects (or the legal representatives).

If a subject can not understand the contents of the informed consent, the study subjects will be included only after obtaining the informed consent through the subject's legitimate representatives.

If it is impossible to obtain the consent of the subject in advance and the subject's legitimate representatives are not present, then the enrollment of such subjects such procedures should be clearly described in the protocol, while the IEC's positive comment and suggestions on protection of the rights and health of the subjects shall also be recorded. In addition, the legitimate representative of the subject should be notified as soon as possible and provid their consent.

**13.4 Confidentiality of individual materials**

This study only collects and handles the indispensable subject data concerning the efficacy, safety, quality and usage.

In collection and use of these data, the confidentiality should be sufficiently guaranteed and the laws and regulations that protect the subject's privacy should be conformed to

The sponsor will ensure that:

- The process of data collection is fair and lawful;
- The purpose of data collection is specific, explicit and legitimate; and no further processing inconsistent with the purpose, in a manner contrary to these purposes, will be done for the data;
- The data collected is adequate, relevant and not excessive with respect to research purposes, and the data unrelated to the purpose of the study will not be collected;
- The collected data is accurate, and will be updated to date if necessary.

Before collecting personal data, the investigators will obtain the consent of the subjects. This consent also includes the transmission and transfer of the data to other institutions and countries.

The subjects are entitled to have an access to the personal materials through the investigators, and may ask them to modify the errors or the incomplete data. As for such requirements, proper responses should be given after their nature, the trial status and relevant laws are properly considered. The nature of such requirements, the trial status and relevant laws and regulations are properly considered.

Proper technical steps and management measures must be adopted to protect the subjects’ personal information from being obtained and publicized without the authorization, to ensure that no accident, illegal destruction, and accidental loss and change will occur. During the entire study, the personnel from the sponsor that are entitled to check the subject personal materials will keep them confidential.

**14 Management requirements**

**14.1 Protocol amendment**

All protocol amendments must be dated and signed, and then issued by the sponsor. The execution cannot be performed before the IEC/IRB is approved, unless under the condition that the purpose is to avoid the existing risk of the subjects, or just the changes on the study logistics or administration (such as typo errors, inconsistency).

During the study, no protocol deviation should be found. In case of any protocol deviation (PD), the investigator should correspondingly handle the PD in time. The reasons and the details of the PD should be recorded in the case report form and the original medical records; the protocol deviation table should be preserved together with the case report forms in the institution and the sponsor.

**14.2 Subject identification and screening logs**

The investigators agree to fill in the subject identity so that the identity of the subjects can be easily recognized during and after the trial. The monitors will verify the completion of his document.

The subject identity is a confidential document, and the investigators should preserve it into the trial site file folds. This registration table should not be duplicated in order to guarantee the confidentiality of the subject identity. All the reports and letters related to the study will adopt the initials and the number to identify the subjects.

The investigators should complete the subject screening recording tables, which record all the doctors that examined the subject, and the doctors determine whether he/she is a qualified subject.

**14.3 Completion of electronic Case Report Form**

This study will adopt electronic Data Collection (EDC) to perform the data collection and management. All data related to the study must be recorded in theelectronic Case Report Form (eCRF) supplied by the sponsor. The investigators must fill in the eCRF in time after the visit, unless any testing results which can not be obstained immediately. Thus, it can be ensured the information on the eCRF will reflect the updated observation results of the subjects.

The investigators must guarantee that all information recorded into eCRFs is exact and accurate.

All eCRF recording, correction and modification must be completed by the investigators or their authorized personnel in the trial sites. All data by researchers of the research centers carried out online via EDC system entry, all data questioned (Query) are sent through the EDC system online by researchers to answer online.

After the completion of data cleaning, the database will be locked.

**14.4 Data quality assurance**

The procedures that need to be adopted to guarantee the data accuracy and reliability include: before the initiation of the study, the qualified investigators and the suitable research units are selected to learn the study content together with the investigators and the related personnel.

During the study, the monitors of the cosponsor (the specified CRO) will monitor the trial on a regular basis. The cosponsor will guide the researchers how to fill in the eCRFs. The monitors of the cosponsor will lon in the EDC system and review the completeness and accuracy of the written eCRFs. The inconsistency in all eCRFs with the original data must be corrected by the investigators or their authorized personnel according to the appropriate manners

**14.5 Monitoring**

The cosponsor will visit the site regularly as needed. The monitor of the cosponsor will record the auditing dates on the audit record sheet, which will be preserved in the research units. The first monitoring after the initiation of the study will be carried out soon after the subjects are included. During the monitoring, the monitors will verify the consistency of the data recorded into the eCRFs and the data in the original medical records in the research units. It is necessary to confirm the nature and preservation site of original documents to guarantee that the investigators are aware of the site of all original data that need to be recorded in the eCRFs and the monitors of the sponsor can be accessible to these data so that they can verify them. If what is preserved is the electronic records, the monitors and the investigators will discuss the methods of verification.and the basic requirement is that the source documentation should at least include: subject identity recognition, inclusion qualification, correct informed consent process, follow-up visit date, implementation of the protocol, records of efficacy and safety indexes, recording and tracing of all adverse events, accompanying medications, date of study completion and reasons. The specific requirements of the original records shall be discussed with the investigators before the start of the study.

To check the consistency of the data in eCRF and that in the original records, the monitor of the cosponsor should be allowed to access all the original data. The monitor will discuss the findings during checking the original records against the eCRF with the researchers.

The sponsor wish that related investigators be present during the monitoring, the original data are accessible, and an appropriate site can provide the related documents of the monitors to read and study. The clinical study monitors will regularly exchange with the investigators about the feedback message related the proceeding of the study.

**14.6 Study completion / discontinuation**

**Study completion**

After the completion of the last follow-up visit of the last subject, the sponsor should be informed and the completion can be considered as the end of the study. The sponsor will notify all the institutions of the ending time of the study and the continuation of the study after the time must be approved by the sponsor, which can be implemented without any amendment.

**Study discontinuation**

The sponsor reserves the right to discontinue the rights of the research units to continue the study at any time. The reasons for the sponsor to discontinue the study/prematurely shut down the site include but are not limited to:

• The number of the enrolled patients has reached the predefined requirement;

• The investigators cannot conform to the protocol or GCP guiding principles；

• The investigators cannot recruit sufficient subjects.

**14.7 Inspection and audit**

The representatives of clinical quality assurance departments (QA) of the cosponsor may pay a visit to the research units to examine whether the trial conforms to the regulations and company policies. During this examination, it is required that all study records including the original medical records be checked, but the right of privacy of the subjects will be respected. Prior to the visit, the investigators should be notified in advance so that there will be sufficient time for proper preparations.

Any pharmaceutical administration department can carry out the similar examinations, and, if the investigators learn that the pharmaceutical administration institutions will inspect some research unit, they should immediately notify the sponsor.

**14.8 Use and publication of information**

All unpublicized information provided by the sponsor to the investigators and any data produced during the study is confidential and exclusive to the sponsor. The investigators agree to keep the information confidential, and the information will be used to complete this study and will not serve for other purposes without the written consent of the sponsor.

The investigators should understand that the information obtained during this study will be used for the further development of the drug; therefore, the information may be provided to other clinical investigators or pharmaceutical administration departments. The investigators have the obligation to provide all the data obtained from this trial to the sponsor for its use.

The study results will be released in the form of clinical study report (CSR)and it includes the data of all participating units. All data and materials originating from the trial and including benefits with patent protection belong to the possession of the sponsor, and it should be stipulated that the Guangdong Techpool Pharmaceutical Stock Co., Ltd (except the following publications written by the investigators) be the author or the owner of the copyright. If the investigators wish to publicize the information in the study, it is necessary to provide the original manuscript to the sponsor for its review 60 days before the submission or lecturing. Arrangement will be made to accelerate the review of the abstracts, posters or other publicity materials.

In case of the issues with regard to the scientific preciseness or the regulation compliance, the sponsor will discuss these problems with the investigators. The sponsor will not mandatorily require the modification on the content and has no right to hide the materials. The investigators should consider the entirety of the multi-center study and can issue the single-center materials only under the following circumstances: the articles that integrate site results have been publicized; all study sites are at the status of completion, abandonment or 12 months after being terminated; the sponsor has confirmed that the multi-center study results will not be publicized. The authorship of the published article on this study will be based on the guidlines,such as the requirements in the "Uniform Requirements for Manuscripts Submitted to Biomedical Journals"; in these regulations, the signed authors must have substantial contribution on the the study design, data analysis and interpretation, provide essential modifications for the article and finally approve the publication of the article.

With the approval of the sponor and the Scientific Recommendation Commission, the data collected in this study may be merged with data from other areas for investigation and analysis in the future. In accordance with applicable laws and regulations, the patients information in the study shall be confidentially managed.

**15 Reference**

[1] Writing group for guidelines on treatment of acute ischemic stroke, Cerebrovascular Disease Team, Neurology branch of Chinese Medical Association. "Guidelines on diagnosis and treatment of acute ischemic stroke in China 2014. Chinese Journal of Neurology, 2010, 43(2): 146-153.

[2] Rao Ming-Li. Guidelines on cerebrovascular disease prevention and treatment. Apoplexy and Nervous Disease October, 2005, 122:5.

[3] Hacke W, Kaste M, Bluhmki E, et al. Thrombolysis with anteplase 3 to 4.5 hours after acute ischemic stroke. N Engl J Med, 2008, 359(13): 1317-1329.

[4] The collaboration group of key research projects in national "Ninth Five-Year Plan". Intravenous thrombolysis with urokinase for acute cerebral infarctions. Chinese Journal of Neurology, 2002, 35: 210-213.

[5] Xydas T, Georgantopoulos C, Bethanis D, et al. Thrombolysis for Acute Ischemic Stroke: a New Paradigm. Hospital Chronicles, 2012, Volume 7, Supplement 1: 77–80.

[6] Jin H, Zhu S, Wei JW, et al. Factors associated with prehospital delays in the presentation of acute stroke in urban China. Stroke. 2012, 43(2): 362-370.

[7] Group of the China Quality Evaluation of Stroke Care and Treatment). Treatment of acute ischemic stroke in China. Chinese Journal of Neurology, 2009, 42(4): 223-228.

[8] Yang B, Shi J, Chen X, et al. Efficacy and safety of therapies for acute ischemic stroke in China: a network meta-analysis of 13289 patients from 145 randomized controlled trials. PLoS One. 2014, 9(2): e88440.

[9] Zhao LiXia, Liu AnChang, Liu XiangHong, et al. Systematic evaluation of ligustrazine in treatment of acute ischemic stroke. Chinese Journal of Gerontology, 2012, 32(15): 3146-3148.

[10] Roger VL, Go AS, Lloyd-Jones DM, et al. Heart disease and stroke statistics–2011 update: a report from the American Heart Association. Circulation, 2011, 123(4):e18-e209.

[11] Chang YanPeng, Xie YanMing. Overview of pharmacoeconomic studies on traditional Chinese medicines and western medicines in treatment of stroke. China Journal of Chinese Materia Medica, 2012, 37(23): 3509-3512.

[12] Ceri Phillips. What is a QALY? Published by Hayward Medical Communications, a division of Hayward Group Ltd. 2009.

[13] Shi Zhu, Qiu DongHai, Zheng WeiCheng, et al. A Survey on execution of secondary prevention regimen for patients with recurrent ischemic stroke. Chinese Journal of Nervous and Mental Diseases, 2012, 38 (8): 482-485.

[14] Wu Jing. Defination and calculation of quality-adjusted life years (QALYs). CHINA JOURNAL OF PHARMACEUTICAL ECONOMICS, 2008,13(4):30-35.

[15] Li MingHui, LuoNan. Introduction on application of Chinese version of EuroQol (EQ-5D). CHINA JOURNAL OF PHA RMACEUTICAL ECONOMICS, 2009, 1: 49-57.

**16 Annex**

**Annex 1: NIH Stroke Scale**

| **Examination** | **Score** |
| --- | --- |
| **1a Level of Consciousness**  The investigator must choose a response if a full evaluation is prevented by such obstacles as an endotracheal tube, language barrier, orotracheal trauma/bandages. A 3 is scored only if the patient makes no movement (other than reflexive posturing) in response to noxious stimulation. | 0 = Alert; keenly responsive.  1 = Not alert; but arousable by minor stimulation to obey, answer, or respond.  2 = Not alert; requires repeated stimulation to attend, or is obtunded and requires strong or painful stimulation  3 = Responds only with reflex motor or autonomic effects or totally unresponsive, flaccid, and areflexic. |
| **1b LOC Questions:**  (It is important that only the initial answer be graded and that the examiner not "help" the patient with verbal or non-verbal cues).  The patient is asked the month and his/her age. The answer must be correct - there is no partial credit for being close. Aphasic and stuporous patients who do not comprehend the questions will score 2. Patients unable to speak because of endotracheal intubation, orotracheal trauma, severe dysarthria from any cause, language barrier, or any other problem not secondary to aphasia are given a 1. | 0 = Answers both questions correctly.  1 = Answers one question correctly.  2 = Answers neither question correctly. |
| **1c LOC Commands:**  The patient is asked to open and close the eyes and then to grip and release the non-paretic hand. Substitute another one step command if the hands cannot be used. Credit is given if an unequivocal attempt is made but not completed due to weakness. If the patient does not respond to command, the task should be demonstrated to him or her (pantomime), and the result scored. Patients with trauma, amputation, or other physical impediments should be given suitable one-step commands. | 0 = Answers both questions correctly.  1 = Performs one task correctly.  2 = Performs neither task correctly. |
| **2 Best Gaze:**  Only horizontal eye movements will be tested. Voluntary or reflexive (oculocephalic) eye movements will be scored. If the patient has a conjugate deviation of the eyes that can be overcome by voluntary or reflexive activity, the score will be 1. If a patient has an isolated peripheral nerve paresis (CN III, IV or VI), score a 1. Gaze is testable in all aphasic patients. Patients with ocular trauma, bandages, pre-existing blindness, or other disorder of visual acuity or fields should be tested with reflexive movements, and a choice made by the investigator. Establishing eye contact and then moving about the patient from side to side will occasionally clarify the presence of a partial gaze palsy. | 0 = Normal.  1 = Partial gaze palsy; gaze is abnormal in one or both eyes, but forced deviation or total gaze paresis is not present.  2 = Forced deviation, or total gaze paresis not overcome by the oculocephalic maneuver. |
| **3 Visual:**  Visual fields (upper and lower quadrants) are tested by confrontation, using finger counting or visual threat, as appropriate. Patients may be encouraged, but if they look at the side of the moving fingers appropriately, this can be scored as normal. If there is unilateral blindness or enucleation, visual fields in the remaining eye are scored. Score 1 only if a clear-cut asymmetry, including quadrantanopia, is found. If patient is blind from any cause, score 3. Double simultaneous stimulation is performed at this point. If there is extinction, patient receives a 1, and the results are used to respond to item 11. | 0 = No visual loss.  1 = Partial hemianopia.  2 = Complete hemianopia.  3 = Bilateral hemianopia (blind including cortical blindness). |
| **4 Facial Palsy:**  Ask – or use pantomime to encourage – the patient to show teeth or raise eyebrows and close eyes. Score symmetry of grimace in response to noxious stimuli in the poorly responsive or non-comprehending patient. If facial trauma/bandages, orotracheal tube, tape or other physical barriers obscure the face, these should be removed to the extent possible. | 0 = Normal.  1 = Minor paralysis (flattened nasolabial fold, asymmetry on smiling).  2 = Partial paralysis (total or near-total paralysis of lower face).  3 = Complete paralysis of one or both sides (absence of facial movement in the upper and lower face). |
| **5 Motor Arm:**  The limb is placed in the appropriate position: extend the arms (palms down) 90 degrees (if sitting) or 45 degrees (if supine). Drift is scored if the arm falls before 10 seconds. The aphasic patient is encouraged using urgency in the voice and pantomime, but not noxious stimulation. The examiner can lift the patient's arm to the required position and encourage the patient to hold on, beginning with the arm of the non-paretic arm. | 0 = No drift; limb holds 90 (or 45) degrees for full 10 seconds.  1 = Drift; limb holds 90 (or 45) degrees, but drifts down before full 10 seconds; does not hit bed or other support.  2 = Some effort against gravity; limb cannot get to or maintain (if cued) 90 (or 45) degrees, drifts down to bed, but has some effort against gravity.  3 = No effort against gravity; limb falls.  4 = No movement.  9 = Amputation or joint fusion, explain:____________ |
| **6 Motor Leg:**  The limb is placed in the appropriate position: hold the leg at 30 degrees (always tested supine). Drift is scored if the leg falls before 5 seconds. The aphasic patient is encouraged using urgency in the voice and pantomime, but not noxious stimulation. The examiner can lift the patient's arm to the required position and encourage the patient to hold on, beginning with the arm of the non-paretic leg. | 0 = No drift; leg holds 30-degree position for full 5 seconds.  1 = Drift; leg falls by the end of the 5-second period but does not hit bed.  2 = Some effort against gravity; leg falls to bed by 5 seconds, but has some effort against gravity.  3 = No effort against gravity; leg falls to bed immediately.  4 = No movement.  9 = Amputation or joint fusion, explain:____________ |
| **7 Limb Ataxia:**  This item is aimed at finding evidence of a unilateral cerebellar lesion. Test with eyes open. In case of visual defect, ensure testing is done in intact visual field. The finger-nose-finger and heel-shin tests are performed on both sides, and ataxia is scored only if present out of proportion to weakness. Ataxia is absent in the patient who cannot understand or is paralyzed. In case of blindness, test by having the patient touch nose from extended arm position. Only in the case of amputation or joint fusion, the examiner should record the score as untestable (UN), and clearly write the explanation for this choice. | 0 = Absent.  1 = Present in one limb.  2 = Present in two limbs.  9 = Amputation or joint fusion, explain:____________ |
| **8 Sensory:**  Sensation or grimace to pinprick when tested, or withdrawal from noxious stimulus in the obtunded or aphasic patient. Only sensory loss attributed to stroke is scored as abnormal and the examiner should test as many body areas (arms [not hands], legs, trunk, face) as needed to accurately check for hemisensory loss. A score of 2, “severe or total sensory loss,” should only be given when a severe or total loss of sensation can be clearly demonstrated. Stuporous and aphasic patients will, therefore, probably score 1 or 0. The patient with brainstem stroke who has bilateral loss of sensation is scored 2. If the patient does not respond and is quadriplegic, score 2. Patients in a coma (item 1a=3) are automatically given a 2 on this item. | 0 = Normal; no sensory loss.  1 = Mild-to-moderate sensory loss; patient feels pinprick is less sharp or is dull on the affected side; or there is a loss of superficial pain  2 = Severe to total sensory loss; patient is not aware of being touched in the face, arm, and leg. |
| **9 Best Language:**  A great deal of information about comprehension will be obtained during the preceding sections of the examination. For this scale item, the patient is asked to describe what is happening in the attached picture, to name the items on the attached naming sheet and to read from the attached list of sentences. Comprehension is judged from responses here, as well as to all of the commands in the preceding general neurological exam. If visual loss interferes with the tests, ask the patient to identify objects placed in the hand, repeat, and produce speech. The intubated patient should be asked to write. The patient in a coma (item 1a=3) will automatically score 3 on this item. The examiner must choose a score for the patient with stupor or limited cooperation, but a score of 3 should be used only if the patient is mute and follows no one-step commands. | 0 = No aphasia; normal.  1 = Mild-to-moderate aphasia: some obvious loss of fluency or facility of comprehension, without significant limitation on ideas expressed or form of expression.  2 = Severe aphasia: all communication is through fragmentary expression; great need for inference, questioning, and guessing by the listener. Range of information that can be exchanged is limited; listener carries burden of communication.  3 = Mute, global aphasia; no usable speech or auditory comprehension. |
| **10 Dysarthria:**  If patient is thought to be normal, an adequate sample of speech must be obtained by asking patient to read or repeat words from the attached list. If the patient has severe aphasia, the clarity of articulation of spontaneous speech can be rated. Only if the patient is intubated or has other physical barriers to producing speech, the examiner should record the score as untestable (UN), and clearly write an explanation for this choice. Do not tell the patient why he or she is being tested. | 0 = Normal.  1 = Mild-to-moderate dysarthria; patient slurs at least some words and, at worst, can be understood with some difficulty.  2 = Patient's speech is so slurred as to be unintelligible in the absence of or out of proportion to any dysphasia, or is mute/anarthric.  9= Intubated or other physical barrier, explain:_____________________________ |
| **11 Neglect:**  Sufficient information to identify neglect may be obtained during the prior testing. If the patient has a severe visual loss preventing visual double simultaneous stimulation, and the cutaneous stimuli are normal, the score is normal. If the patient has aphasia but does appear to attend to both sides, the score is normal. Through detecting the patient's ability to identify the cutaneous sensation and visual stimulus that occur simultaneously to both left and right sides, whether the patient neglects it is judged. The standard map is presented to the patient and he/she is required to describe. The doctor encourages the patient to see the map carefully and identify the characteristics of both the left and right sides in the map. If the patient fails to identify partial contents of one side of the map, it is defined as abnormal. Then, the doctor asks to the patient to close the eyes, and examines the bilateral cutaneous sensations through detecting the upper or lower limb pinprick sensation. In case that the patient has unilateral sensation neglect, it is defined as abnormal. | 0 = No abnormality.  1 = Visual, tactile, auditory, spatial, or personal inattention or extinction to bilateral simultaneous stimulation in one of the sensory modalities.  2 = Profound hemi-inattention or extinction to more than one modality; does not recognize own hand or orients to only one side of space. |

Source:

Brott TG, Adams HP Jr, Olinger CP, et al. Measurements of acute cerebral infarction: a clinical examination scale. Stroke, 1989, 20: 864-870.

Annex 2: mRS Scoring criteria

| **0** | No symptoms at all |
| --- | --- |
| **1** | No significant disability despite symptoms; able to carry out all usual duties and activities |
| **2** | Mild disability; unable to carry out all previous activities, but able to look after own affairs without assistance |
| **3** | Moderate disability; requiring some help, but able to walk without assistance |
| **4** | Moderate disability; unable to walk without assistance and unable to attend to own bodily needs without assistance |
| **5** | Severe disability; bedridden, incontinent and requiring constant nursing care and attention |
| **6** | Death |

Source:

van Swieten JC, Koudstaal PJ, Visser MC, et al. Interobserver agreement for the assessment of handicap in stroke patients. Stroke, 1988, 19(5):604-607.

**Annex 3: EQ-5D scale**

By placing a tick in one box in each group below, please indicate which statements best describe your own health state today. (√)。

**ACTIONS**

| I have no problems in walking about | □ |
| --- | --- |
| I have some problems in walking about | □ |
| I am confined to bed | □ |
| **Self-care** |  |
| I have no problems with self-care | □ |
| I have some problems washing or dressing myself | □ |
| I am unable to wash or dress myself | □ |
| **Usual Activities** (e.g. work, study, housework, family or leisure activities) |  |
| I have no problems with performing my usual activities | □ |
| I have some problems with performing my usual activities | □ |
| I am unable to perform my usual activities | □ |
| **Pain/Discomfort** |  |
| I have no pain or discomfort | □ |
| I have moderate pain or discomfort | □ |
| I have extreme pain or discomfort | □ |
| **Anxiety** (such as nervous, worrying, uneasy)/**Depression** |  |
| (Such as lack of interest in doing things, no fun, no energy) |  |
| I am not anxious or depressed | □ |
| I am moderately anxious or depressed | □ |
| I am extremely anxious or depressed | □ |

| To help people say how good or bad a health state is, we have drawn a scale (rather like a thermometer) on which the best state you can imagine is marked 100 and the worst state you can imagine is marked.  We would like you to indicate on this scale how good or bad your own health is today, in your opinion. Please do this by drawing a line from the box below to whichever point on the scale indicates how good or bad your health state is today.  Your own health state today | Best imaginable health state |
| --- | --- |
|  | 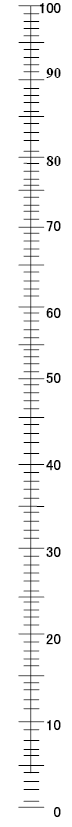 |
|  |  |
|  | Worst imaginable health state |

Source:Li MingHui, LuoNan. Introduction on application of Chinese version of EuroQol (EQ-5D). CHINA JOURNAL OF PHA RMACEUTICAL ECONOMICS, 2009, 1: 49-57.

**Appendix 4: Glasgow coma scale (GCS)**

Eye opening

4 points - Spontaneous

3 points - To sound

2 points - Localizes painful stimuli

1 point - None

- Verbal repsonse

5 points - Oriented, conserves normally

4 points - Confused, disoriented

3 points Utters inappropriate words

2 points Incomprehensible sounds

1 point - Makes no sounds

movement

6 points - Obey commands

5 points - Localizes painful stimuli

4 points - Flexion / Withdrawal to painful stimuli

3 points - Abnormal flexion to painful stimuli (decorticate response)

2 points - Extension to painful stimuli (decerebrate response)

1 point - Makes no movements

Source:

Teasdale G, Jennett B. Assessment of coma and impaired consciousness. [Lancet,](http://www.ncbi.nlm.nih.gov/pubmed/4136544/) 1974, 2(7872): 81-84.

**Appendix 5: Mini-Mental State Examination (MMSE)**

| Item | | Score | | | | | |
| --- | --- | --- | --- | --- | --- | --- | --- |
| Orientation  (10 points) | 1. What is the year?  What is the season?  What is the month?  What is the date?  What day is today? |  |  |  |  | 1  1  1  1  1 | 0  0  0  0  0 |
|  | 2. Where are your province?  Where are your county (district)?  Where are your town (streetrict)?  Where are we hospital?  Where are we floor? |  |  |  |  | 1  1  1  1  1 | 0  0  0  0  0 |
| Memory  3 points | 3. I will name three objects. Then I will ask you repeat the 3 objects and remember them after I have said them, and I will asked you again (1 point for each correct answer, altogether 3 points) |  |  | 3 | 2 | 1 | 0 |
| Attention and Calculation  5 points | 4. 100-7=？ Serial 5's. (93, 86, 79, 72, 65) 1point for each correct answer, altogether 5 points。 If this answer is wrong and the next answer is right, just record a fault once.) | 5 | 4 | 3 | 2 | 1 | 0 |
| Recall  3 points | 5. Ask for the objects repeated above. |  |  | 3 | 2 | 1 | 0 |
| Language  9 points | 6. Naming ability  Show a watch, and ask the patient to name it. Show a pen, and ask the patient to name it. |  |  |  |  | 1  1 | 0  0 |
|  | 7. Repeating ability  I will say a sentence, please repeat it once clearly (No ifs, ands, or buts”) |  |  |  |  | 1 | 0 |
|  | 8. Reading ability  Read and obey the following: CLOSE YOUR EYES |  |  |  |  | 1 | 0 |
|  | 9. Follow a 3-stage command:  I will give you a paper and follow my commands “Take a paper in your right hand, fold it in half by hoth hands, and put it on your left leg.” (one point for each activity, altogether 3 points) |  |  | 3 | 2 | 1 | 0 |
|  | 10. Writing ability  Ask the patient to write a complete sentence |  |  |  |  | 1 | 0 |
|  | 11. Structure ability 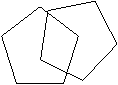  ((show the pattern) copy the pattern shown! |  |  |  |  | 1 | 0 |

Source:

Zhang Mingyuan, Elena Yu, He Yanling. Investigational tools for dementia epidemiology and applications Shanghai Psychiatry, 1995, 7 (Suppl): 1-62.
